# Supplementary figures and images for: Human albumin enhances the pathogenic potential of Candida glabrata on vaginal epithelial cells
Source: PLoS Pathog. 2021 Oct 28;17(10):e1010037. doi: 10.1371/journal.ppat.1010037 (PMC8577789; doi:10.1371/journal.ppat.1010037)

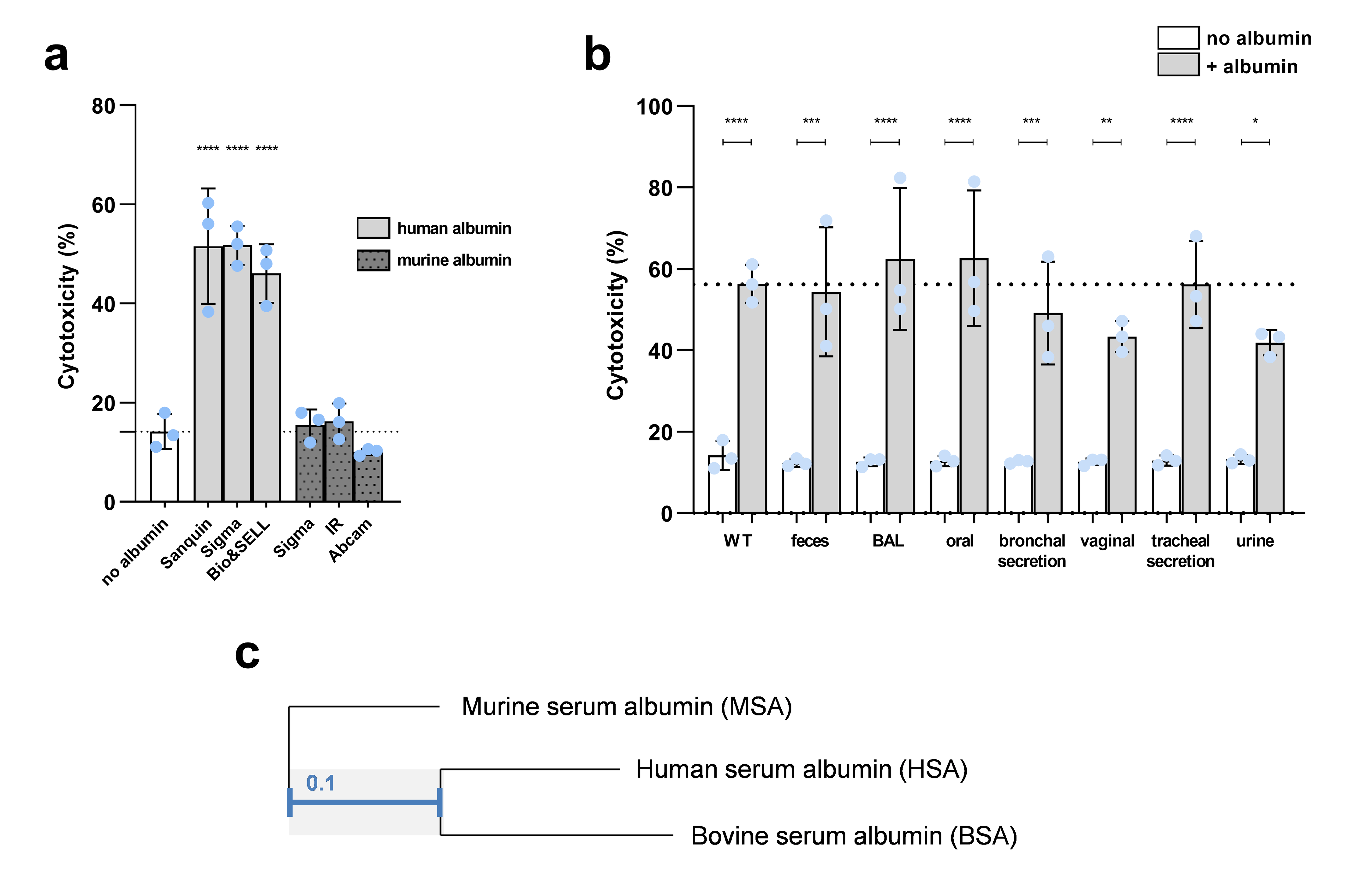

Supplement: S1 Fig — (a) Damage of A-431 cells infected with C. glabrata with human or murine albumin from three different manufacturers (Sanquin Plasma Products B.V., Albuman; Bio & Sell; Sigma–for human albumin; Sigma; Innovative Research (IR); Abcam–for murine albumin). The dotted line represents the damage from A-431 cells in medium only. (b) Damage of A-431 cells infected with different C. glabrata clinical strains isolated from various anatomical sites, with or without albumin. The dotted line represents the damage from A-431 cells infected with wild type (WT) C. glabrata in the presence of albumin. (c) Dendrogram showing clustering of human (ID: Q56G89), bovine (ID: P02769.4) and murine (ID: P07724.3) albumin protein sequences using BLASTP tool provided by NCBI. The alignment of albumin sequences is provided in S1 File, obtained using Jalview. All values are presented as mean ± SD. Damage was recorded by measuring the lactate dehydrogenase activity in the supernatant and presented as percentage of a full lysis control (A-431 treated with Triton X-100). Albumin was always used at a 5 mg/mL concentration. One-way ANOVA (a) or two-way ANOVA (b) were used to calculate statistically significant differences. Statistical significance is indicated as: *, p ≤ 0.05; **, p ≤ 0.01; ***, p ≤ 0.001; ****, p ≤ 0.0001. Abbreviation: BAL—bronchoalveolar lavage. (TIF) [file ppat.1010037.s001.tif]

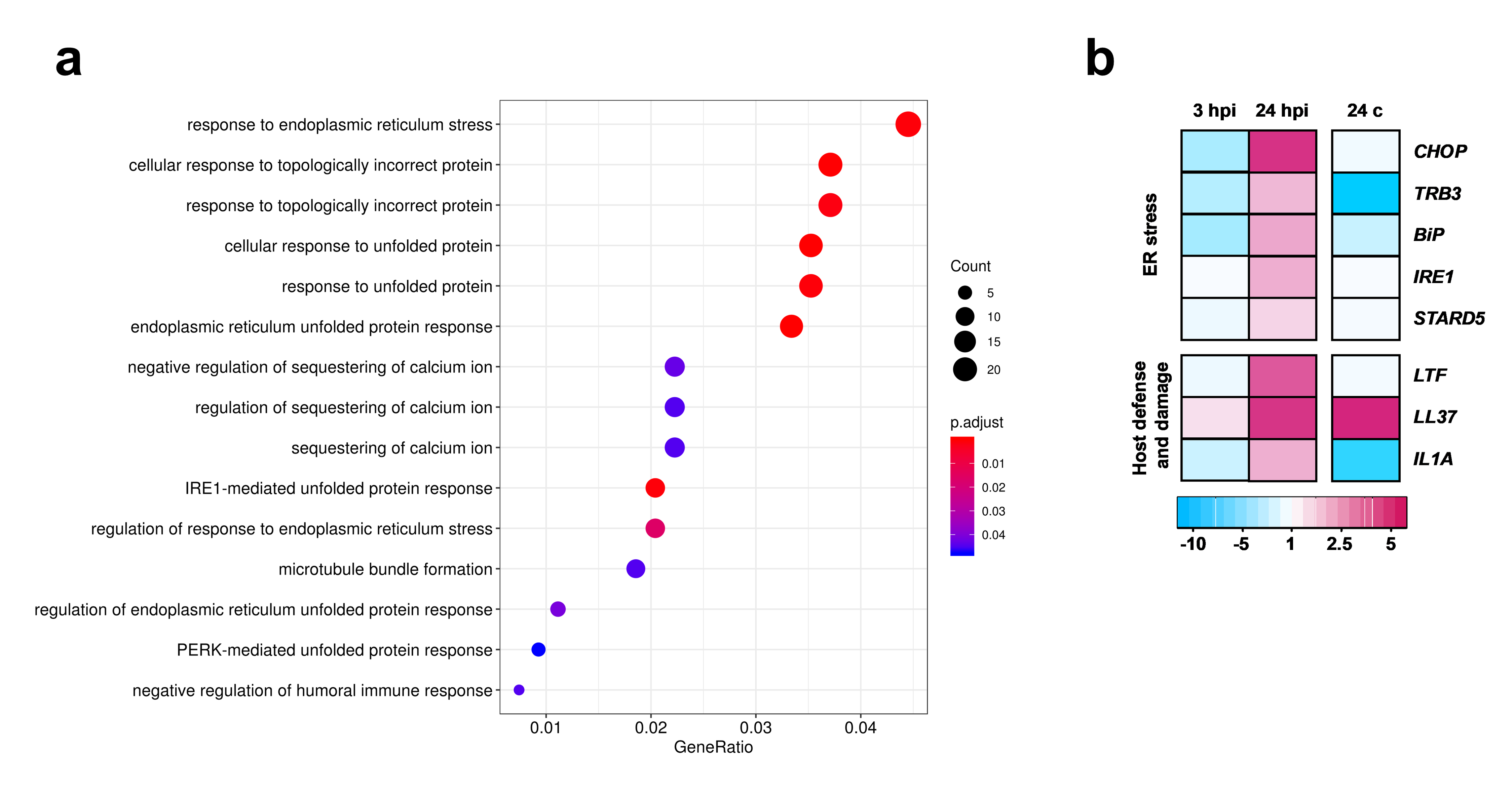

Supplement: S2 Fig — (a) Expression of adhesin and putative adhesin C. glabrata genes at 3 and 24 hpi and 24 h control (24 c) in the presence of albumin, derived from RNA-Seq data (see Material and Methods). Presented values are expressed as log2 fold changes of expression compared to fungal cells in medium only at the time point 0. (b) Principal component analysis (PCA) bi-plot of all analysed fungal and (c) human samples (n = 3 for each time point; technical replicates are merged into individual samples). Control samples represent the transcriptional response of C. glabrata only or the host only to medium with albumin at 0 and 24 hours. Labels of the samples correspond to internal sample identifiers. (d) Expression of human genes involved in starvation at 3 and 24 hpi and 24 h control (24 c) in the presence of albumin, derived from RNA-Seq data (see Material and Methods). Presented values are expressed as log2 fold changes of expression compared to host cells in medium only at the time point 0. (TIF) [file ppat.1010037.s002.tif]

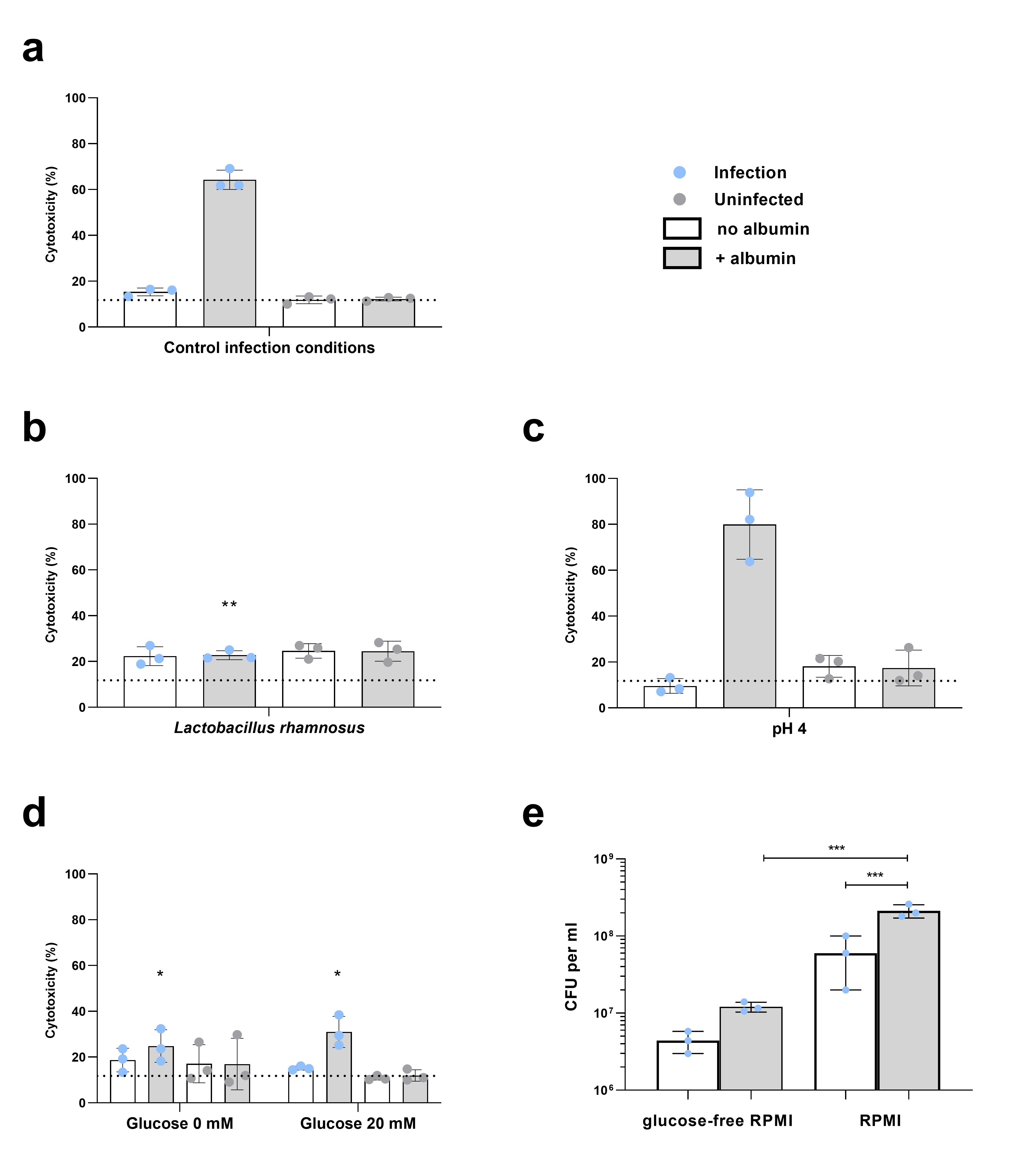

Supplement: S3 Fig — (a) Damage of A-431 infected with C. glabrata with or without albumin in standard conditions (RPMI pH 7–7.4, 11 mM glucose). (b) Damage of Lactobacillus rhamnosus-colonized A-431 cells infected with C. glabrata with or without albumin. (c) Damage of A-431 infected with C. glabrata with or without albumin at pH 4. (d) Damage of A-431 infected with C. glabrata with or without albumin in RPMI with increased glucose (20 mM) availability or without glucose (0 mM). (e) C. glabrata growth 24 h post infection of A-431 cells with or without human or murine albumin in culture medium with or without glucose. All values are presented as mean ± SD. Damage was recorded by measuring the lactate dehydrogenase activity in the supernatant and presented as percentage of a full lysis control (A-431 treated with Triton-X-100). The dotted line represents damage from uninfected A-431 cells. Albumin was always used at a 5 mg/mL concentration. One-way ANOVA was used to calculate statistically significant differences between control infection experiment (a) and other conditions (b-d). Statistical significance is indicated as: *, p ≤ 0.05; **, p ≤ 0.01; ***, p ≤ 0.001. (TIF) [file ppat.1010037.s003.tif]
