# Supplementary material for: Human albumin enhances the pathogenic potential of Candida glabrata on vaginal epithelial cells
Source: PLoS Pathog. 2021 Oct 28;17(10):e1010037. doi: 10.1371/journal.ppat.1010037 (PMC8577789; doi:10.1371/journal.ppat.1010037)
Supplement: S1 File — Comparison of human (ID: Q56G89), bovine (ID: P02769.4) and murine (ID: P07724.3) albumin protein sequences using Jalview. (HTML) [file ppat.1010037.s005.html]

Launch in Jalview  

{"seqs":[{"name":"Human\_albumin/1-608","start":1,"end":608,"id":"295473395","seq":"MKWVTFISLLFLFSSAYSRGVFRRDAHKSEVAHRFKDLGEENFKALVLIAFAQYLQQCPFEDHVKLVNEVTEFAKTCVADESAENCDKSLHTLFGDKLCTVATLRETYGEMADCCAKQEPERNECFLQHKDDNPNLPRLVRPEVDVMCTAFHDNEETFLKKYLYEIARRHPYFYAPELLFFAKRYKAAFTECCQAADKAACLLPKLDELRDEGKASSAKQRLKCASLQKFGERAFKAWAVARLSQRFPKAEFAEVSKLVTDLTKVHTECCHGDLLECADDRADLAKYICENQDSISSKLKECCEKPLLEKSHCIAEVENDEMPADLPSLAADFVESKDVCKNYAEAKDVFLGMFLYEYARRHPDYSVVLLLRLAKTYETTLEKCCAAADPHECYAKVFDEFKPLVEEPQNLIKQNCELFEQLGEYKFQNALLVRYTKKVPQVSTPTLVEVSRNLGKVGSKCCKHPEAKRMPCAEDYLSVVLNQLCVLHEKTPVSDRVTKCCTESLVNRRPCFSALEVDETYVPKEFNAETFTFHADICTLSEKERQIKKQTALVELVKHKPKATKEQLKAVMDDFAAFVEKCCKADDKETCFAEEGKKLVAASQAALG","order":1},{"name":"Bovine\_albumin/1-607","start":1,"end":607,"id":"890869891","seq":"MKWVTFISLLLLFSSAYSRGVFRRDTHKSEIAHRFKDLGEEHFKGLVLIAFSQYLQQCPFDEHVKLVNELTEFAKTCVADESHAGCEKSLHTLFGDELCKVASLRETYGDMADCCEKQEPERNECFLSHKDDSPDLPKL-KPDPNTLCDEFKADEKKFWGKYLYEIARRHPYFYAPELLYYANKYNGVFQECCQAEDKGACLLPKIETMREKVLASSARQRLRCASIQKFGERALKAWSVARLSQKFPKAEFVEVTKLVTDLTKVHKECCHGDLLECADDRADLAKYICDNQDTISSKLKECCDKPLLEKSHCIAEVEKDAIPENLPPLTADFAEDKDVCKNYQEAKDAFLGSFLYEYSRRHPEYAVSVLLRLAKEYEATLEECCAKDDPHACYSTVFDKLKHLVDEPQNLIKQNCDQFEKLGEYGFQNALIVRYTRKVPQVSTPTLVEVSRSLGKVGTRCCTKPESERMPCTEDYLSLILNRLCVLHEKTPVSEKVTKCCTESLVNRRPCFSALTPDETYVPKAFDEKLFTFHADICTLPDTEKQIKKQTALVELLKHKPKATEEQLKTVMENFVAFVDKCCAADDKEACFAVEGPKLVVSTQTALA","order":2},{"name":"Murine\_albumin/1-608","start":1,"end":608,"id":"649841779","seq":"MKWVTFLLLLFVSGSAFSRGVFRREAHKSEIAHRYNDLGEQHFKGLVLIAFSQYLQKCSYDEHAKLVQEVTDFAKTCVADESAANCDKSLHTLFGDKLCAIPNLRENYGELADCCTKQEPERNECFLQHKDDNPSLPPFERPEAEAMCTSFKENPTTFMGHYLHEVARRHPYFYAPELLYYAEQYNEILTQCCAEADKESCLTPKLDGVKEKALVSSVRQRMKCSSMQKFGERAFKAWAVARLSQTFPNADFAEITKLATDLTKVNKECCHGDLLECADDRAELAKYMCENQATISSKLQTCCDKPLLKKAHCLSEVEHDTMPADLPAIAADFVEDQEVCKNYAEAKDVFLGTFLYEYSRRHPDYSVSLLLRLAKKYEATLEKCCAEANPPACYGTVLAEFQPLVEEPKNLVKTNCDLYEKLGEYGFQNAILVRYTQKAPQVSTPTLVEAARNLGRVGTKCCTLPEDQRLPCVEDYLSAILNRVCLLHEKTPVSEHVTKCCSGSLVERRPCFSALTVDETYVPKEFKAETFTFHSDICTLPEKEKQIKKQTALAELVKHKPKATAEQLKTVMDDFAQFLDTCCKAADKDTCFSTEGPNLVTRCKDALA","order":3}],"appSettings":{"globalColorScheme":"% Identity","webStartUrl":"https://www.jalview.org/services/launchApp","application":"Jalview","showSeqFeatures":"false","version":"2.11.1.4"},"seqGroups":[],"alignAnnotation":[],"svid":"1.0","seqFeatures":[]}

  

xml version="1.0"?


Human\_albumin/1-608Bovine\_albumin/1-607Murine\_albumin/1-608ConservationQualityConsensusOccupancyHuman\_albumin/1-608Bovine\_albumin/1-607Murine\_albumin/1-608ConservationQualityConsensusOccupancyHuman\_albumin/1-608Bovine\_albumin/1-607Murine\_albumin/1-608ConservationQualityConsensusOccupancyHuman\_albumin/1-608Bovine\_albumin/1-607Murine\_albumin/1-608ConservationQualityConsensusOccupancyMKWVTFISLLFLFSSAYSRGVFRRDAHKSEVAHRFKDLGEENFKALVLIAFAQYLQQCPFEDHVKLVNEVTEFAKTCVADESAENCDKSLHTLFGDKLCTVATLRETYGEMADCCAKQEPERNECFLQHKDDNPNLPRLVRPEVDVMCTAFHDNEETFLKKYLYEIARRHPYFYAPELLFFAKRYKAAFTEMKWVTFISLLLLFSSAYSRGVFRRDTHKSEIAHRFKDLGEEHFKGLVLIAFSQYLQQCPFDEHVKLVNELTEFAKTCVADESHAGCEKSLHTLFGDELCKVASLRETYGDMADCCEKQEPERNECFLSHKDDSPDLPKL-KPDPNTLCDEFKADEKKFWGKYLYEIARRHPYFYAPELLYYANKYNGVFQEMKWVTFLLLLFVSGSAFSRGVFRREAHKSEIAHRYNDLGEQHFKGLVLIAFSQYLQKCSYDEHAKLVQEVTDFAKTCVADESAANCDKSLHTLFGDKLCAIPNLRENYGELADCCTKQEPERNECFLQHKDDNPSLPPFERPEAEAMCTSFKENPTTFMGHYLHEVARRHPYFYAPELLYYAEQYNEILTQ\*\*\*\*\*\*+5\*\*8958\*\*9\*\*\*\*\*\*\*98\*\*\*\*9\*\*\*96\*\*\*\*85\*\*+\*\*\*\*\*\*8\*\*\*\*7\*7999\*8\*\*\*9\*9\*9\*\*\*\*\*\*\*\*\*\*447\*9\*\*\*\*\*\*\*\*\*7\*\*5978\*\*\*9\*\*99\*\*\*\*4\*\*\*\*\*\*\*\*\*\*\*8\*\*\*\*9\*7\*\*48-9\*96779\*74\*948557\*759\*\*8\*9\*\*\*\*\*\*\*\*\*\*\*\*\*99\*57\*647888                                                                                                                                                                                               MKWVTFISLLFLFSSAYSRGVFRRDAHKSEIAHRFKDLGEEHFKGLVLIAFSQYLQQCPFDEHVKLVNEVTEFAKTCVADESAANCDKSLHTLFGDKLC+VA+LRETYGEMADCC+KQEPERNECFLQHKDDNP+LP+L+RPE+++MCT+FK+NE+TF+GKYLYEIARRHPYFYAPELLYYA++YN++FTECCQAADKAACLLPKLDELRDEGKASSAKQRLKCASLQKFGERAFKAWAVARLSQRFPKAEFAEVSKLVTDLTKVHTECCHGDLLECADDRADLAKYICENQDSISSKLKECCEKPLLEKSHCIAEVENDEMPADLPSLAADFVESKDVCKNYAEAKDVFLGMFLYEYARRHPDYSVVLLLRLAKTYETTLECCQAEDKGACLLPKIETMREKVLASSARQRLRCASIQKFGERALKAWSVARLSQKFPKAEFVEVTKLVTDLTKVHKECCHGDLLECADDRADLAKYICDNQDTISSKLKECCDKPLLEKSHCIAEVEKDAIPENLPPLTADFAEDKDVCKNYQEAKDAFLGSFLYEYSRRHPEYAVSVLLRLAKEYEATLECCAEADKESCLTPKLDGVKEKALVSSVRQRMKCSSMQKFGERAFKAWAVARLSQTFPNADFAEITKLATDLTKVNKECCHGDLLECADDRAELAKYMCENQATISSKLQTCCDKPLLKKAHCLSEVEHDTMPADLPAIAADFVEDQEVCKNYAEAKDVFLGTFLYEYSRRHPDYSVSLLLRLAKKYEATLE\*\*644\*\*48\*\*7\*\*+948997868\*\*89\*\*99\*8\*9\*\*\*\*\*\*\*8\*\*\*8\*\*\*\*\*\*6\*\*6\*9\*8\*98\*\*8\*\*\*\*\*\*57\*\*\*\*\*\*\*\*\*\*\*\*\*\*\*9\*\*\*\*9\*9\*\*58\*\*\*\*\*76\*\*9\*\*\*\*7\*8\*\*+8\*\*\*5\*49\*48\*\*6+8\*\*\*8\*779\*\*\*\*\*6\*\*\*\*8\*\*\*6\*\*\*\*\*8\*\*\*\*9\*8\*69\*\*\*\*\*\*5\*\*8\*\*\*                                                                                                                                                                                               CCQAADK+ACLLPKLD++REK+LASSARQRLKCAS+QKFGERAFKAWAVARLSQ+FPKAEFAEVTKLVTDLTKVHKECCHGDLLECADDRADLAKYICENQDTISSKLKECCDKPLLEKSHCIAEVE+D+MPADLP+LAADFVEDKDVCKNYAEAKDVFLG+FLYEYSRRHPDYSVSLLLRLAK+YEATLEKCCAAADPHECYAKVFDEFKPLVEEPQNLIKQNCELFEQLGEYKFQNALLVRYTKKVPQVSTPTLVEVSRNLGKVGSKCCKHPEAKRMPCAEDYLSVVLNQLCVLHEKTPVSDRVTKCCTESLVNRRPCFSALEVDETYVPKEFNAETFTFHADICTLSEKERQIKKQTALVELVKHKPKATKEQLKAVMDECCAKDDPHACYSTVFDKLKHLVDEPQNLIKQNCDQFEKLGEYGFQNALIVRYTRKVPQVSTPTLVEVSRSLGKVGTRCCTKPESERMPCTEDYLSLILNRLCVLHEKTPVSEKVTKCCTESLVNRRPCFSALTPDETYVPKAFDEKLFTFHADICTLPDTEKQIKKQTALVELLKHKPKATEEQLKTVMEKCCAEANPPACYGTVLAEFQPLVEEPKNLVKTNCDLYEKLGEYGFQNAILVRYTQKAPQVSTPTLVEAARNLGRVGTKCCTLPEDQRLPCVEDYLSAILNRVCLLHEKTPVSEHVTKCCSGSLVERRPCFSALTVDETYVPKEFKAETFTFHSDICTLPEKEKQIKKQTALAELVKHKPKATAEQLKTVMD7\*\*\*358\*34\*\*87\*857873\*\*9\*\*7\*\*9\*8\*\*979\*7\*\*\*\*5\*\*\*\*++\*\*\*\*7\*8\*\*\*\*\*\*\*\*\*\*88\*9\*\*9\*\*89\*\*75\*\*56\*9\*\*7\*\*\*\*\*79\*\*89\*9\*\*\*\*\*\*\*\*98\*\*\*\*\*84\*\*\*7\*\*\*\*\*\*\*\*67\*\*\*\*\*\*\*4\*5477\*\*\*\*8\*\*\*\*\*797\*9\*\*\*\*\*\*\*\*8\*\*9\*\*\*\*\*\*\*3\*\*\*\*8\*\*9                                                                                                                                                                                               KCCA+ADPHACY+TVFDEFKPLVEEPQNLIKQNCDLFEKLGEYGFQNALLVRYT+KVPQVSTPTLVEVSRNLGKVGTKCCT+PE++RMPC+EDYLS+ILNRLCVLHEKTPVSE+VTKCCTESLVNRRPCFSALTVDETYVPKEF+AETFTFHADICTLPEKEKQIKKQTALVELVKHKPKAT+EQLKTVMDDFAAFVEKCCKADDKETCFAEEGKKLVAASQAALGNFVAFVDKCCAADDKEACFAVEGPKLVVSTQTALADFAQFLDTCCKAADKDTCFSTEGPNLVTRCKDALA8\*86\*997\*\*5\*5\*\*98\*\*84\*\*46\*\*74775\*\*+                                   DFAAFVDKCCKADDKETCFA+EGPKLV+++Q+ALA111191190191192191192382381382383382383573572573574573574608607608
