# Supplementary material for: Human albumin enhances the pathogenic potential of Candida glabrata on vaginal epithelial cells
Source: PLoS Pathog. 2021 Oct 28;17(10):e1010037. doi: 10.1371/journal.ppat.1010037 (PMC8577789; doi:10.1371/journal.ppat.1010037)
Supplement: Attachment — Submitted filename: Cover letter_Response to the comments_final.docx [file ppat.1010037.s008.docx]

Dear Editor,

We would like to thank you and the referees for the detailed and very constructive comments. To address the raised issues by the Guest Editor and both referees, we have performed additional experiments and analyses as requested. Specifically, we extended the infection experiments by testing additional conditions (increased iron supply, different glucose concentrations, the presence of lactobacilli and an acidic pH), which provided new depth to our study and strengthens our results and conclusions. We also extended the analysis of our transcriptional data to better understand the role of albumin presence in the context of iron-related genes in *C. glabrata* and ER stress-related genes in epithelial cells.

As a result, we provide additional data and further discussion as requested, which we believe has improved the manuscript. Additionally, we now provide a “Source data” file that contains all the data used to generate graphs in our manuscript, as per editorial guidelines.

Our replies to the referees’ comments are below, in blue:

Comments by the Guest Editor:

Dr. Hube:

Your manuscript has been reviewed by the editorial board and two independent experts in the field. Both reviewers felt the manuscript has considerable merit. Please consider all the reviewer's comments in your rebuttal and revision. Pay particular attention to reviewer 1 who suggests some additional experimentation for confirmation of your findings. There are also additional issues to consider for comment/discussion in the revised manuscript. In addition to the review, I also suggest that you consider a different term in the title for 'multiplies'. This term implies a quantitative value of sorts. A more qualitative term would be more appropriate.

Authors’ response: We want to thank the editorial board for reviewing our work and providing constructive suggestions. Regarding the title, we used “multiplies” to also refer to the “quantative” multiplication of *C. glabrata*, as we observed increased growth. However, in order to avoid potential confusion, we changed the word “multiplies” to “enhances” as suggested.

Second, due to the potential role of glucose in the human albumin-associated effects on vaginal epithelial cells, you should consider including a few comments on this effect relative to diabetes. Women with diabetes tend to have higher prevalence of VVC or RVVC caused by *C. glabrata*. This could be in part due to the higher presence of glucose available.

Authors’ response: We agree that diabetes is definitely an important risk factor for VVC and RVVC. Mimicking diabetes using *in vitro* models is rather simplified to altering glucose levels, as it is difficult to recapitulate *in vitro* the physiological changes (e.g. increased glycosylation, hyperinflammatory responses) that persist for years in patients. Nevertheless, we performed additional experiments to evaluate the role of different glucose concentrations in our model (see also the reply to suggestions by Reviewer 2 below).

We observed that albumin was unable to enhance *C. glabrata*-induced damage and proliferation in the absence of glucose. This suggests that *C. glabrata* requires glucose as a carbon source to sustain its enhanced growth and damage potential in the presence of albumin.

However, when we increased glucose availability in the culture medium we also observed decreased potential of albumin to enhance *C. glabrata-*induced damage. We believe that the reduced damage in this scenario may be the result of reduced glucose starvation of epithelial cells and therefore lower cytotoxicity. We have included these data in Supplementary Fig. 3 and the results section (lines 392-399). Furthermore, we commented on this and potential implications in diabetes in the discussion part (lines 491-499).

Responses to Reviewer's Questions

**Part I – Summary**

Reviewer #1: Pekmezovic et al. address the effect of human serum albumin on the interaction of C. glabrata with vaginal epithelial cells. They find that human and bovine – but not mouse – albumin promote proliferation of C. glabrata in the presence of vaginal epithelial cells, as well as damage to the epithelial cells by C. glabrata. Albumin increases adherence of C. glabrata to epithelial cells. The cytotoxicity requires direct contact to the epithelial cells, but the increased C. glabrata proliferation does not: it also occurs in a transwell setup. Protease inhibitors, and inhibition of albumin uptake by epithelial cells, abolish the increased cell damage, suggesting that albumin uptake and processing by the cells mediates increased sensitivity to C. glabrata. Protease inhibitors also abolish the increased C. glabrata proliferation.
Expression profiling in C. glabrata indicated an increase in oxidative stress resistance genes, and indeed C. glabrata cells grown in the presence of epithelial cells with albumin are more resistant to H2O2. A screen for C. glabrata mutants that cause less epithelial cell damage in spite of having no reduced growth capacity identified HAP5, encoding an iron-regulated transcription factor. Transferrin and BPS abolish the albumin-stimulated growth and the damage potential of C. glabrata, leading the authors to the suggest that iron is responsible for the albumin-mediated stimulation of C. glabrata proliferation and damage to epithelial cells.

Evaluation: the question of how to best model virulence of pathogens in vitro is key in the field of microbial pathogenesis. Therefore, the identification of human albumin as a specific host factor involved in virulence of a fungal pathogen is an important finding. This is a well-written paper in which the authors show through a series of convincing experiments that the mechanism by which albumin increases C. glabrata-induced damage to epithelial cells involves epithelial cell uptake and processing of albumin. A possible involvement of iron release or uptake, while being an attractive hypothesis based on the phenotype of the hap5 mutant, is less convincing at this point (see comments below).

Authors’ response: We thank Reviewer 1 for the detailed review of our study, encouraging comments and providing constructive feedback to strengthen our conclusions regarding the role of iron metabolism.

Reviewer #2: The manuscript by Pekmezovic et al describes a careful work on the role of human (and bovine) albumin on the pathogenesis of the important human fungal pathogen, Candida glabrata. It is well known, that C. glabrata causes quite frequently candidiasis in humans (actually it is the second or third most common cause of this disease), surprisingly however this species is often almost avirulent in different in vivo and in vitro model systems. The authors investigated the biological background of this contradiction and found that human albumin (digested by the human epithelial cells) can increase the growth, adhesion, and damage-causing capacity of C. glabrata cells. The screening of a deletion library revealed that Hap5, an important regulator of the iron homeostasis, is a key regulator of the albumin-dependent virulence enhancement. The manuscript is very well written and easy to follow.

Authors’ response: We thank Reviewer 2 for the detailed review our manuscript and positive feedback.

**Part II – Major Issues: Key Experiments Required for Acceptance**

Reviewer #1: 1) Role of iron: in Fig. 6b, 6c, the authors interpret the abolition of albumin-stimulated cytotoxicity and C. glabrata growth by iron chelators as evidence that this stimulation involves iron supply. However, an alternative explanation is that albumin relaxes another, unknown limiting factor for proliferation and epithelial cell damage, whereas addition of chelators impose iron with holding as a new limiting factor.

Furthermore, the induction seen in Fig. 6d of an iron starvation response in C. glabrata exposed to epithelial cells with albumin (as evidenced by upregulation of iron uptake genes such as FTR1, SIT1, FTH1) suggests that iron is withheld, rather than supplied, under these conditions, contradicting the notion that improved growth can be attributed to an increased iron supply.

Thus, the model that iron supply is driving the increased C. glabrata proliferation and cytotoxicity in the presence of albumin is not well supported by the data. The notion that iron supply is a limiting factor to begin with could be corroborated by testing the effect of direct iron addition to the RPMI medium.

Authors’ response: This is a fair pointed raised by the reviewer. Indeed, we agree that the alternative explanation could also be a possible mechanism that occurs in our model. Therefore, we discuss this alternative explanation in the revised manuscript (line 452-453).

To test the role of increased iron availability, we performed additional experiments where we supplemented the medium with ferrous sulfate (Fig. 6d). We observed that increased iron availability in the form of ferrous sulfate similarly increased *C. glabrata* damage potential as albumin, even in the absence of albumin. This observation justifies that increasing iron availability to *C. glabrata* is important for its pathogenic potential and induction of host cell cytotoxicity*.*

Regarding the induction of above-mentioned genes, these are induced upon iron starvation to increase the pathogen’s capacity to acquire iron. Since the proteins encoded by these genes are involved in iron uptake, it is conceivable that these proteins would also be required to deal with increased iron availability. For example, the study by Riedelberger *et al.* (Riedelberger *et al.* 2020, *Cell Host Microbe* 27(3):454-466.e8. DOI: 10.1016/j.chom.2020.01.023), showed that enhanced iron accumulation in the phagolysosome leads to iron acquisition by *C. glabrata via* the Sit1/Ftr1 iron transporter system, which facilitated intracellular proliferation. We discussed this in the revised manuscript (lines 465-467).

We believe that investigating the role of iron uptake genes under iron restriction and iron excess, requires further in-depth investigation, especially in the context of the host where iron can potentially be acquired from a variety of sources.

2) Induction of Hap5-dependent iron-regulated genes by albumin: to my understanding, the experiment shown in Fig 6d doesn’t address this (i.e., there is no comparison +/- albumin). In order to make the point that it is albumin addition that induces the transcriptional response, incubation of C. glabrata in medium with epithelial cells, with vs. without albumin, should be compared, rather than comparing medium + epithelial cells + albumin vs. medium alone. I.e., the effects of albumin should be tested by comparing conditions +/– albumin, everything else remaining equal.

Authors’ response: We would like to thank the reviewer for pointing out this issue. We have performed additional experiments and data analysis and in the revised manuscript we provide a new heatmap (now Fig.6e). This revised heatmap shows the transcriptional response of both strains +/– albumin during the infection and in medium alone and demonstrates a clear induction of Hap5-dependent iron-regulated genes by albumin.

Likewise for Fig. 7: the gene expression in epithelial cells seems to compare only +/- C. glabrata in the presence of albumin, not infection with C. glabrata +/- albumin. An analysis of the effect of albumin alone on the cells is shown after 24h, however the comparison is to t0. Comparison to an identical 24h culture without albumin would have been more adequate.

Authors’ response: As stated above, the revised heatmap in the Fig.7b now includes data for both conditions, including the identical 24h culture without albumin. The revised heatmap still highlights the induction of an ER-stress and host defense genes in response to *C. glabrata* infection in the presence of albumin, with the exception of LL37, which seems to be specifically induced by host cells upon exposure to albumin irrespective of the infection status.

Reviewer #2: (No Response)

**Part III – Minor Issues: Editorial and Data Presentation Modifications**
Please use this section for editorial suggestions as well as relatively minor modifications of existing data that would enhance clarity.

Reviewer #1: ll. 115-116: what’s the MOI in albumin-free medium?

Authors’ response: Unless otherwise stated, the MOI used in our study was always 1 (as indicated in the Materials and Methods section). For clarity, we included this information in the main text (Results) of the revised manuscript (line 113).

ll. [300-302](tel:300302): “Albumin can bind iron and was also demonstrated to deliver iron to C. albicans via the CFEM hemophore relay network (21,33)”: this should be qualified, because albumin was shown to bind and deliver heme, not free iron. There is no indication of specific iron binding by serum albumin to my knowledge (the iron binding protein in serum is transferrin, as mentioned later). Furthermore, there is no evidence for the existence of a CFEM hemophore system in C. glabrata.

Authors’ response: Thank you for noticing this issue, we agree that these statements were not precise enough and could be misleading. We have revised the wording (lines 291-295).

Fig. 1c: the similarity tree of the three albumins isn’t very informative. However the availability of a non-stimulating albumin, MSA, might enable the authors to test their suggestion that HSA and BSA function by binding and supplying iron: iron binding could be directly tested (e.g., retention of radioactive iron by membrane-bound albumin), with MSA providing a negative control.

Authors’ response: We appreciate this suggestion. The difference in the ability to bind iron could be one of the explanations why MSA and HSA/BSA have different effects. We started to perform colorimetric assays to quantify the amount of iron bound by HSA and MSA and we could see a slightly higher binding by HSA, however, the read values were very low. One of the reasons could be that the concentration of iron (ferrous sulfate) we used for incubation with HSA/MSA was not appropriate (0.6 ng/µl), but it was hard to choose the representative iron concentration that would match the one present in our model, as it is more complex and iron is available in different compartments and cells. Furthermore, it might be that proteolytic activity by the host cells generates peptides that are binding more iron and testing the intact proteins would not show the same effect. We would like to show these data here for the reviewer only, but we would not include this in the manuscript, because of the preliminary nature as the assay requires further optimization.

We would like to point out that the discrepancy between mouse and human albumin may not solely rely on the capacity to bind iron, but also on uptake and processing by host cells. This has been discussed in the Discussion section of the manuscript (line 553-556). We are, however, very keen on further exploring the differences between human and murine albumin in a follow up study, as we appreciate that this could be a line of explanation regarding the avirulence of *C. glabrata* in mice.

**Figure R1. The level of bound iron by HSA and MSA.** HSA/MSA (0.5 mg/ml) were incubated for one hour in RPMI with or without 0.6 ng/µl of ferrous sulfate. Protein solutions were then washed using 30K columns (Amicon Ultra-0.5 mL Centrifugal Filters, Millipore) to remove the media with unbound ferrous sulfate. The total iron was quantified using Iron Assay Kit (catalog number MAK025, Sigma).

Reviewer #2: I only have some minor suggestions and questions:

1. The normal vaginal pH is between 3.8 and 4.5, the authors used RPMI for the cytotoxicity assays, which has a higher pH level (7.2-8.3 depending of the manufacturer) did the authors considered this factor, since the pH level of the environment can influence the virulence related properties?
2. The vagina has a complex and well-studied microbial community, factor that can influence C. glabrata virulence and pathogenesis in the presence of the human albumin predominantly composed of Lactobacillus species. Did the authors considered this microbial components of the vagina as a potential? I suggest at least mention this important aspect in the discussion.

Authors’ response: The reviewer raises an important point that the low pH in the vagina can drastically impact the interactions that we studied in our model at neutral pH. Furthermore, we agree that microbes, and in particular lactobacilli, play a key role in the balance between commensalism and pathogenicity. We, therefore, performed additional experiments using several physiologically relevant conditions for the vaginal niche (pH 4, addition of lactobacilli and different glucose concentrations).

- We observed that albumin increased the pathogenic potential of *C. glabrata* independent of the pH.
- Furthermore, similar to the protective role of the microbiota against VVC, colonization of epithelial cells by *L. rhamnosus* was protective against *C. glabrata-*induced damage, even in the presence of albumin.
- We observed that albumin was unable to enhance *C. glabrata*-induced damage and proliferation in absence of glucose. This suggests that *C. glabrata* requires glucose as a carbon source to sustain its enhanced growth and damage potential in the presence of albumin. When we increased glucose availability in the culture medium this also decreased the potential of albumin to enhance *C. glabrata-*induced damage. We believe that the reduced damage may be the result of reduced glucose starvation and therefore lower cytotoxicity (see response to the Guest Editor).

We have included these data in Supplementary Fig.3 and the results section (lines 377-399). Furthermore, we discussed the implications of these findings in the discussion (lines 491-499).

3. The increased expression of the adhesion related genes at the early timepoint of the albumin exposure is an interesting finding, did the authors further tested the adhesion ability of the C. glabrata cells in the presence of human albumin? The increased expression of genes of adhesins and putative adhesins caused indeed an increased attachment to the host cells?

Authors’ response: This is a valid point. In line 105 we already described the influence of albumin on adhesion at early time points: “Apart from damage, *C. glabrata* exhibited also increased adhesion to vaginal epithelial cells (Fig 1c) ...” We also tried to quantify the adhesion in the presence of albumin at the later time points (3 and 6 hpi) (when the higher level of mRNA of these genes should have been led to increased protein levels of the corresponding adhesion genes). However, the dramatic growth-promoting effect of albumin made it impossible to accurately calculate adhesion rate compared to the non-albumin control. The reason is that it is not possible to distinguish whether increased rates of attached cells detected in the presence of albumin is a result of increased adherence or simply because there are more fungal cells present (due to increased growth).

4. During the screen of the mutant library, some deletion mutants showed an increased damage capacity in compare to the control, is there any speculation about the reason of this results?

Authors’ response: The reviewer appropriately addresses the point of deletion mutants that showed an increased damage capacity. As recently described by Siscar-Lewin *et al.* (Siscar-Lewin *et al.* 2019 *Virulence* 10(1):935-947. DOI: 10.1080/21505594.2019.1688753), we speculate that these genes could act as antivirulence genes. Therefore, their loss could contribute to the increased virulence, as we observed in our model by elevated damage capacity.

While revising your submission, please upload your figure files to the Preflight Analysis and Conversion Engine (PACE) digital diagnostic tool, [https://pacev2.apexcovantage.com](https://pacev2.apexcovantage.com/). PACE helps ensure that figures meet PLOS requirements. To use PACE, you must first register as a user. Then, login and navigate to the UPLOAD tab, where you will find detailed instructions on how to use the tool. If you encounter any issues or have any questions when using PACE, please email us at [figures@plos.org](mailto:figures@plos.org).

Authors’ response: We have checked the figure quality with PACE

Data Requirements:

Please note that, as a condition of publication, PLOS' data policy requires that you make available all data used to draw the conclusions outlined in your manuscript. Data must be deposited in an appropriate repository, included within the body of the manuscript, or uploaded as supporting information. This includes all numerical values that were used to generate graphs, histograms etc.. For an example see here on PLOS Biology: <http://www.plosbiology.org/article/info%3Adoi%2F10.1371%2Fjournal.pbio.1001908#s5>.

Authors’ response: We uploaded all source data as supplementary information.
